# A miR-129-5P/ARID3A Negative Feedback Loop Modulates Diffuse Large B Cell Lymphoma Progression and Immune Evasion Through Regulating the PD-1/PD-L1 Checkpoint

**DOI:** 10.3389/fcell.2021.735855

**Published:** 2021-10-27

**Authors:** Weili Zheng, Guilan Lai, Qiaochu Lin, Mohammed Awal Issah, Haiying Fu, Jianzhen Shen

**Affiliations:** Fujian Provincial Key Laboratory on Hematology, Fujian Medical Center of Hematology, Fujian Institute of Hematology, Clinical Research Center for Hematological Malignancies of Fujian Province, Fujian Medical University Union Hospital, Fuzhou, China

**Keywords:** miR-129-5p, ARID3a, PD-1/PD-L1, lymphoma, ABC-type

## Abstract

The activated B cell (ABC) and germinal center B cell (GCB) subtypes of diffuse large B cell lymphoma (DLBCL) have different gene expression profiles and clinical outcomes, and miRNAs have been reported to play important roles in tumorigenesis, progression, and metastasis. This study aimed to explore the differentially expressed miRNAs and target genes in the two main subtypes of DLBCL. Hub miRNAs were identified by constructing a regulatory network, and *in vitro* experiments and peripheral blood samples of DLBCL were used to explore the functions and mechanisms of differential miRNAs and mRNAs. Differentially expressed miRNAs and genes associated with the two DLBCL subtypes were identified using GEO datasets. Weighted gene co-expression network analysis shows that one gene module was associated with a better prognosis of patients with the GCB subtype. Through the construction of a regulatory network and qPCR verification of clinical samples and cell lines, miR-129-5p was identified as an important differential miRNA between the ABC and GCB subtypes. The negative relationship between miR-129-5p and ARID3A in DLBCL was confirmed using luciferase reporter assays. Overexpression of miR-129-5p and knockdown of ARID3A inhibited the proliferation of SU-DHL-2 (ABC-type) cells and promoted their apoptosis through the JAK and STAT6 signaling pathways. In addition, inhibition of miR-129-5p and overexpression of ARID3A promoted the proliferation and reduced apoptosis of DB and SU-DHL-6 (GCB-type) cells. Inhibition of miR-129-5p and overexpression of ARID3A in DB and SU-DHL-6 promoted immune escape by increasing PD-L1 expression, which was transcriptionally activated by ARID3A. In conclusion, we showed for the first time that the mir-129-5P/ARID3A negative feedback loop modulates DLBCL progression and immune evasion by regulating PD-1/PD-L1.

## Introduction

Diffuse large B cell lymphoma (DLBCL) is the most common form of malignant lymphoma, accounting for 25–35% of all non-Hodgkin lymphomas (Miao et al., 2019). R-CHOP chemo-immunotherapy has improved outcomes for DLBCL patients; however, approximately 40% of them relapse or fail to respond to treatment (Li M. et al., 2019). With the development of high-throughput technologies, the genomic profile of DLBCL has been widely characterized. DLBCL is classified into two molecular subtypes based on cell of origin (COO), germinal center B cell (GCB)-like, and activated B cell (ABC)-like (Alizadeh et al., 2000), which is also associated with different clinical outcomes. It has been reported that the ABC subtype is related to inferior chemotherapeutic responses and clinical outcomes compared to the GCB subtype (Fu et al., 2008) following R-CHOP therapy. Therefore, various studies have identified miRNA- (Yang et al., 2018) or lncRNA-focused (Zhou et al., 2017) prognostic biomarkers or signatures between different COO subtypes.

MicroRNAs, comprised of 18–25 nucleotides, widely participate in many biological processes to regulate gene expression by specifically combining to mRNAs, promoting their degradation, and eventually reducing translation (Bruch et al., 2019). Dysregulation of miRNAs has been found to play a crucial role in tumorigenesis, tumor progression, and metastasis by negatively regulating tumor-suppressive protein-coding genes (Wong et al., 2018). In addition, several miRNAs modulate the sensitivity of tumor cells to anticancer drugs, which affects treatment response and prognosis. For example, miR145-3p enhances bortezomib sensitivity in multiple myeloma by promoting apoptosis and autophagy (Wu et al., 2019). Additionally, miR-34a is associated with a superior response to doxorubicin in DLBCL (Marques et al., 2016). Hence, miRNAs have been evaluated as novel diagnostic and prognostic biomarkers in various malignancies, including DLBCL (Marchesi et al., 2018). Moreover, some studies have shown that miRNAs can distinguish between GCB and ABC subtypes (Larrabeiti-Etxebarria et al., 2019).

ARID3a/Bright, an AT-rich interacting domain family of DNA-binding proteins, was originally discovered because of its ability to enhance antigen-ABC immunoglobulin gene transcription. ARID3a forms a dimer with DNA through an arid zone or an a/t-rich interaction domain. Overexpression of ARID3a leads to skewing of mature B cell subsets and changes in gene expression patterns of follicular B cells, whereas loss of its function leads to the loss of B1 lineage B cells and defects in hematopoiesis (Nixon et al., 2004). ARID3A expression is tightly regulated, and B cell-restricted and abnormal expression of ARID3A may result in malignancy and proliferative capacity (Puissegur et al., 2012).

Programmed cell death 1 ligand 1(PD-L1), also known as cluster of differentiation 274 or B7 homolog 1 (Butte et al., 2007), plays an important role in tumor progression and survival by evading immune surveillance when interacting with PD-1 to regulate tumor-specific T cells. PD-L1 overexpression in tumor cells confers protection against CD8^+^ cell damage, leading to immune evasion (Freeman et al., 2002). Blocking the PD-1/PD-L1 checkpoint by antibodies is therefore considered an effective method for tumor immunotherapy, including lymphoma (Sun et al., 2018).

In this study, we aimed to screen miRNAs and target genes in two main subtypes of DLBCL through bioinformatics and experiments, and to explore their functions and mechanisms.

## Materials and Methods

### Acquisition of Gene Expression Profiles and Identification of Differentially Expressed Genes

MiRNA (GSE15250) and mRNA (GSE56313 and GSE32918) expression data from DLBCL patients were acquired from the GEO database. Data from 23 ABC-type and 29 GCB-type patients were obtained dataset GSE56313, and data from 80 ABC-type and 120 GCB-type patients were obtained from dataset GSE32918. The miRNA dataset GSE15250, which included data from 20 ABC-type and 20 GCB-type patients, was analyzed. Differentially expressed miRNAs and mRNAs between ABC-type and GCB-type patients were identified using the LIMMA package in R software.

### Weighted Gene Co-expression Network Analysis

After obtaining differentially expressed genes (DEGs) from the GSE32918 database, the expression of these genes was analyzed using the weighted gene co-expression network analysis (WGCNA) package in R software (Langfelder and Horvath, 2008). WGCNA was used to construct a weighted adjacency matrix that expressed the connection strength of gene pairs by calculating Pearson’s correlation. Then, the appropriate soft threshold power β was selected using the scale-free topology criterion and the adjacency matrix was transformed into a topological overlapping matrix. Topological overlapping points were applied to perform hierarchical clustering. Mean linkage hierarchical clustering was applied to generate a clustering tree, and the dendrogram was defined as Module (Langfelder et al., 2008).

### Construction of the Regulatory Network

A regulatory network of the differentially expressed miRNAs and mRNAs was constructed using the miRMap (Vejnar and Zdobnov, 2012), miRanda (Betel et al., 2008), miRDB (Wong and Wang, 2015), TargetScan (Agarwal et al., 2015), and miTarBase (Chou et al., 2018) databases. Only the miRNA-mRNA pairs present in at least two databases were considered significant and were preserved for further investigation. Eventually, cytoscape (Shannon et al., 2003) was used for network visualization.

### Cell Culture

Human B-lymphoma cell lines (ABC subtype) SU-DHL-2, (GCB subtype) DB were obtained from Procell Life (Wuhan, China), and (GCB subtype) SU-DHL-6 were obtained from American Type Culture Collection (ATCC, Manassas, VA, United States), and were grown in RPMI-1640 (Invitrogen, Carlsbad, CA, United States) containing 10% fetal bovine serum (FBS; Gibco, Waltham, MA, United States). Human renal epithelial cells (293T) were gifted by the Fujian Institute of Hematology and grown in DMEM (Invitrogen) containing 10% FBS. All cells were cultured in a humidified incubator at 37°C and 5% CO_2_.

### Cell Transfection

Specific shRNAs and oeRNAs against ARID3A (sh-ARID3A, oe-ARID3A) and sh-NC and oe-NC were obtained using pLVshRNA and pCDH-CMV vector designed by Miaolingbio (P0268, P0684, Wuhan, China). MiR-129-5p mimic/inhibitor and NC mimic/inhibitor were generated by Genechem (Shanghai, China). These plasmids were transfected into DLBCL cells using Lipofectamine 3000.

### Quantitative Real-Time Polymerase Chain Reaction

Total RNA from peripheral blood and cells was extracted by Trizol and purified by chloroform and ethanol. Reverse transcription for miRNA and mRNA based on All-in-One^TM^ miRNA qRT-PCR Detection Kit (QP115, GeneCopoeia, United States) and Revert Aid First Strand cDNA Synthesis Kit (K1621, Thermo Fisher Scientific, United States), respectively. Relative expression levels were detected by FastStart Universal SYBR Green Master (Roche) and Biosystems 7500 Real-Time PCR Systems and calculated by 2−ΔΔCt method. miRNA and mRNA were normalized to U6 and GADPH levels, respectively. The experiment was repeated three times. Primers used for qRT-PCR analysis are listed in Supplementary Table 1.

### Assessment of Cell Proliferation and Apoptosis

Cell counting assays at different times were used to assess cell proliferation ability. Transfected cells were seeded in 24-well plates. Following incubation for 0, 24, 72, or 96 h, homogenized single cell suspension and trypan blue were mixed 1:1. The cells were counted directly under a light microscope with the inclusion of three counting replicates per well. An APC Annexin V Apoptosis Detection kit (Biolegend, San Diego, CA, United States) was used to analyze cells following the manufacturer’s instructions. A BD Accuri C6 flow cytometer was used to analyze the samples.

### *In vitro* Co-culture System and Flow Cytometry

*In vitro* co-culture was performed using a Transwell cell incubator (Millipore Corporation, Billerica, MA, United States). In the co-culture system, lymphoma cells were placed in the upper cavity and immune cells in the lower cavity, with both cells in direct contact. Immune cells were mononuclear cells (including lymphocytes and monocytes) isolated from peripheral blood of healthy volunteers using Ficoll by density gradient centrifugation. After 48-h incubation, the immune cells were centrifuged at 500 × g, 4°C for 5 min. Then, the cells were first blocked with mouse IgG mAb and then surface-stained with anti-CD8 (555369, BD) and anti-PD-1 (367404, Biolegend) antibody at 4°C for 20 min in the dark. The cells were washed twice with FACS and were then loaded for data collection. At least 200,000 events were measured using BD Accuri C6 flow cytometer. An isotype control was used for the antibodies. The data were analyzed using the FlowJo software (TriStar Inc., El Segundo, CA, United States). The gating strategy for the ICS assay is shown in Supplementary Figure 1. These results are representative from six independent experiments.

### Luciferase Report Assay

The pmirGLO dual-luciferase vector (P0198, Miaolingbio, China), including the ARID3A and miR-129-5p mimics, were transfected into 293T cells with Lipofectamine 3000. The PD-L1 promoter WT/Mut was included into the pGL3-basic vector (P0193, Miaolingbio, China) and transfected into 293T cells with oe-ARID3A or oe-NC. Luciferase activity was measured using the Dual-Luciferase Reporter Assay System (E2920, Promega), and a Glomax 96 spectrophotometer was used to detect the fluorescence intensity.

### Western Blot Analysis

Briefly, cells were lysed with RIPA buffer (keyGEN, China) containing protease inhibitors. 20 μg of protein per sample was loaded, run on 10% SDS–polyacrylamide gel electrophoresis, and transferred to a PVDF membrane. After being blocked with 5% bovine serum albumin, membranes were incubated with primary antibodies including PD-L1 (66248, Proteintech), JAK1(3344T, Cell signaling technology), STAT6 (51073, Proteintech) GAPDH (ab8245, Abcam) at 4°C overnight, 1 h of incubation with secondary antibodies and detected by FlourChemE system (Protein Simple).

### Survival Analysis

DLBCL patients were divided into low-and high-expression groups based on their gene expression profiles. Kaplan-Meier survival curves were drawn to demonstrate the relevance between expression of DEGs and overall survival (OS) of patients, which was tested by the log-rank test.

### Statistical Analysis

Student’s *t*-test or one-way analysis of variance were implemented. Pearson correlation analysis was used to determine the relationship between miR-129-5p, ARID3A, and CD8^+^ T cells. Statistical significance was set at *P* < 0.05.

## Results

### Identification of Differentially Expressed Genes Associated With Clinical Molecular Subtypes

**FIGURE 1:**
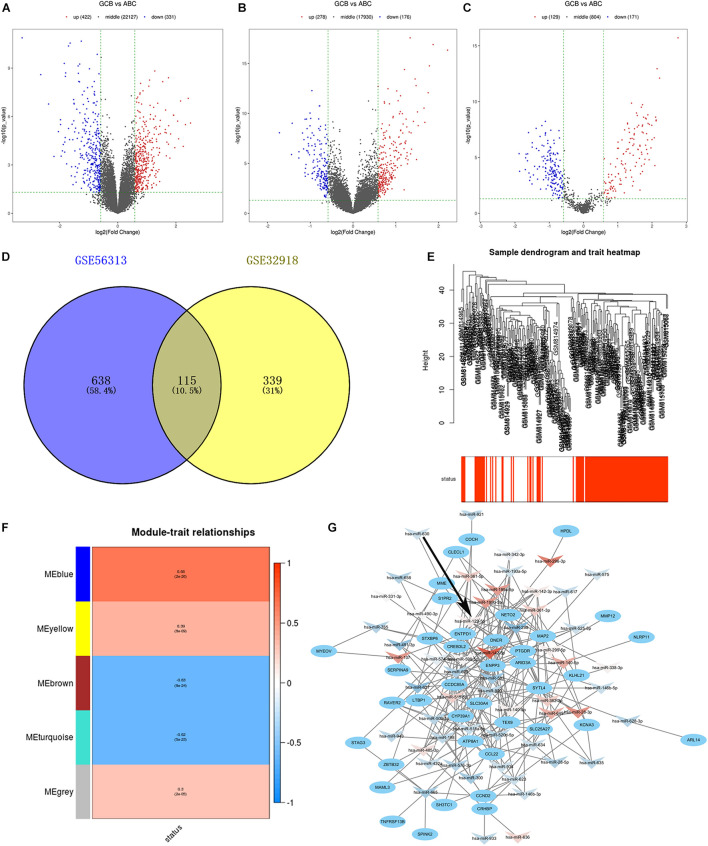
**(A–C)** Volcano plots show gene expression in the GSE56313, GSE32918, and GSE15250 datasets. Red and blue symbols indicate genes that were significantly up- and downregulated, respectively. **(D)** Venn diagrams showing the number of common DEGs between GSE56313 and GSE32918. **(E)** A hierarchical clustering dendrogram is used to arrange DLBCL samples based on GSE32918, with survival outcomes shown at the bottom. **(F)** A module-trait relationship matrix is shown with rows and columns corresponding to survival statue and the module eigengenes, and the correlation and *p*-values are represented in a every box. **(G)** Regulatory network of miRNA-target mRNA pairs in modular intersection genes. The network contained 56 differentially expressed miRNAs (arrow triangle) and 39 target mRNAs (oval). Red and blue arrow triangles indicate genes that were significantly up- and downregulated, respectively, in the GCB subtype.

We compared the gene expression profiles of GSE56313 and GSE32918 datasets and identified DEGs associated with two major clinical molecular subtypes of DLBCL (ABC and GCB). Of these, 331 genes were downregulated and 422 upregulated in the GSE56313 dataset, and 176 genes were downregulated and 278 upregulated in the GSE32918 dataset (Figures 1A,B). The miRNA dataset GSE15250 was also analyzed, finding 171 downregulated and 129 upregulated miRNAs related to different DLBCL subtypes (Figure 1C). By analyzing the intersection of the DEGs, 115 common DEGs were identified, which were named as differentially intersected genes (DIGs) (Figure 1D and Supplementary Table 2).

### Weighted Gene Co-expression Network Analysis Network Construction

We conducted WGCNA based on 454 DEGs and survival data from the GSE32918 dataset. Hierarchical clustering of samples based on Euclidean distance calculated using log10 was converted (Figure 1E). By merging modules with high similarity, we identified five different modules of co-expressed genes that were drawn in different colors (Figure 1F). Among the five modules, the blue module was significantly correlated with good survival status of GCB subtype patients compared to ABC subtype patients, with Pearson *r* = 0.66 (*p* = 2e^–26^). There were 124 genes in the blue module, which are likely associated with a better prognosis of patients of the GCB subtype. These 124 significant genes were intersected with DEGs from the GSE56313 dataset to generated modular intersection genes (MIGs, Supplementary Table 3), which were selected for subsequent analysis.

### Regulatory Networks of Modular Intersection Genes

Construction of regulatory networks of MIGs was performed based on the correlation between differentially expressed miRNAs and target genes using Cytoscape. A total of 21 upregulated miRNAs, 35 downregulated miRNAs, and 39 matched mRNAs were identified in comparison to the GCB and ABC subtypes (Figure 1G and Supplementary Table 4). Among them, hsa-miR-142-5p possessed the most regulatory target genes (*n* = 10). In addition, hsa-miR-199b-5p and hsa-miR-129-5p had nine target genes. Therefore, these miRNAs were considered to be the key miRNA differences between the ABC and GCB subtypes.

### Validation of Differentially Expressed miRNAs for Clinical Subtypes in Diffuse Large B Cell Lymphoma Samples

**FIGURE 2:**
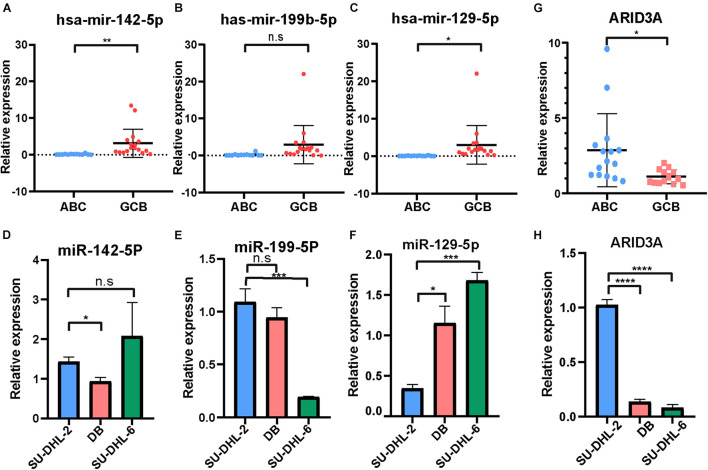
**(A–C)** RT-qPCR showing the expression of hsa-miR-142-5p, hsa-miR-199b-5p, and hsa-miR-129-5p in the ABC and GCB subtypes DLBCL patients.**(D–F)** RT-qPCR showing the expression of hsa-miR-142-5p, hsa-miR-199b-5p, and hsa-miR-129-5p in the SU-DHL-2 (ABC subtype) and DB and SU-DHL-6 (GCB subtype) cell lines. **(G,H)** QRT-PCR showing the expression of ARID3A in the ABC and GCB subtypes DLBCL patients and cell lines, respectively. All data represented mean ± SD from three independent experiments. NS: *P* > 0.05; *P < 0.05; **P < 0.01; ***P < 0.001; ****P < 0.0001.

The expression of the three important miRNAs mentioned above, hsa-miR-142-5p, hsa-miR-199b-5p, and hsa-miR-129-5p, in DLBCL patients with ABC and GCB subtypes was detected using qRT-PCR (Figures 2A–C). Except for hsa-miR-199b-5p, the miRNAs were highly expressed in the GCB subtype compared to the ABC subtype. In addition, the expression of the three miRNAs in the DLBCL cell line was investigated (Figures 2D–F). The results demonstrate that hsa-miR-129-5p was expressed at significantly higher levels in the DB and SU-DHL-6 (GCB subtype) cell lines than in the SU-DHL-2 (ABC subtype). According to the above results, hsa-miR-129-5p has nine target genes, of which ARID3A has been reported to be involved in progression of a variety of tumors (Ma et al., 2017; Dausinas et al., 2020; Tang et al., 2020). To identify the role of ARID3A in DLBCL subtypes, we also explored its expression in the peripheral blood of DLBCL patients and in DLBCL cell lines. Conversely, ARID3A was significantly increased in ABC subtype DLBCL patients and in the SU-DHL-2 cell line, in contrast to the GCB subtype (Figures 2G,H).

### A Negative Regulatory Feedback Loop Between miR-129-5p and ARID3A in Diffuse Large B Cell Lymphoma

**FIGURE 3:**
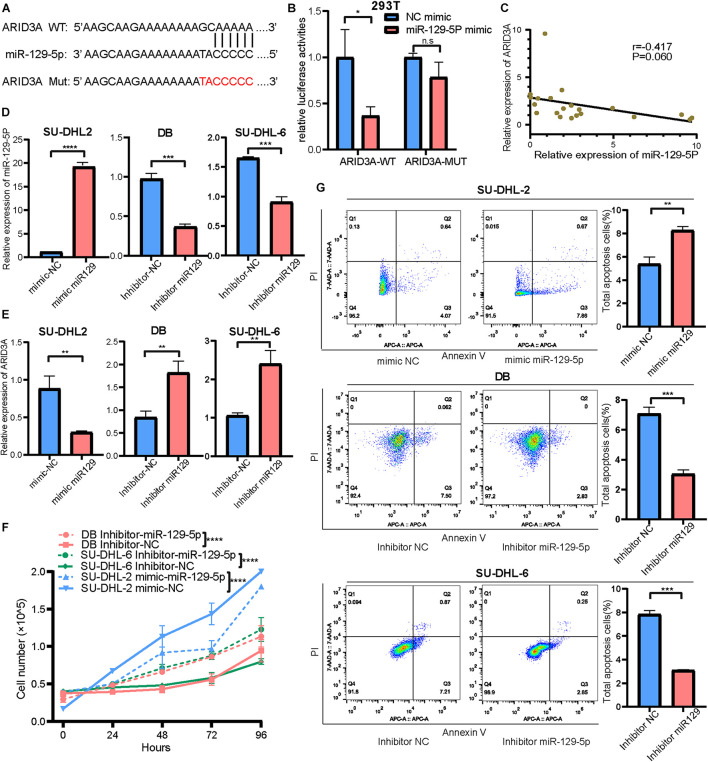
**(A)** Interaction sequences of ARID3A for miR-129-5p binding were acquired from Starbase v3.0 and mutated by altering them with complementary sequences. **(B)** Dual-luciferase reporter gene assays were performed to detect the interaction between ARID3A and miR-129-5p. **(C)** Pearson’s correlation curve showing that ARID3A was negatively correlated with miR-129-5p in DLBCL peripheral blood. **(D)** The inhibition and overexpression of miR-129-5p in DLBCL cells was confirmed with RT-qPCR assays. **(E)** RT-qPCR showing the expression of ARID3A in miR-129-5p-overexpressing SU-DHL-2 cells and miR-129-5p -knockdown DB and SU-DHL-6 cells. **(F)** Cell proliferation ability of transfected cells. Cells were placed in 24-well plates and counted after 0, 24, 72, and 96 h and the cell number were determined with trypan blue exclusion staining. **(G)** Cell apoptosis of SU-DHL-2 with mimic miR-129-5p, DB and SU-DHL-6 with inhibitor miR-129-5p, and negative control cells was measured through flow cytometry analysis. All data represented mean ± SD from three independent experiments. *P < 0.05; **P < 0.01; ***P < 0.001; ****P < 0.0001.

To detect the detailed relationship between miR-129-5p and ARID3A, the site of miR-129-5p for ARID3A was predicted, mutated, and evaluated in dual-luciferase reporter gene assays (Figure 3A). Overexpression of miR-129-5p weakened the luciferase activity of ARID3A WT (wild-type), but did not influence the luciferase activity of ARID3A Mut (mutation), demonstrating that miR-129-5p affects ARID3A at the predicted binding site (Figure 3B). Pearson correlation coefficient analysis shows a negative interaction between miR-129-5p and ARID3A in DLBCL samples, although the difference was not statistically significant (Figure 3C). Furthermore, the reciprocal relationship between miR-129-5p and ARID3A was verified in DLBCL cells. After confirming the inhibition and overexpression of miR-129-5p in DLBCL cells via qRT-PCR (Figure 3D), ARID3A was found to be downregulated in miR-129-5p-overexpressing SU-DHL-2 cells and upregulated in miR-129-5p -knockdown DB and SU-DHL-6 cells (Figure 3E). We conclude that miR-129-5p and ARID3A form a negative regulatory feedback loop in DLBC.

### Effect of miR-129-5p and ARID3A on Diffuse Large B Cell Lymphoma Proliferation and Apoptosis

**FIGURE 4:**
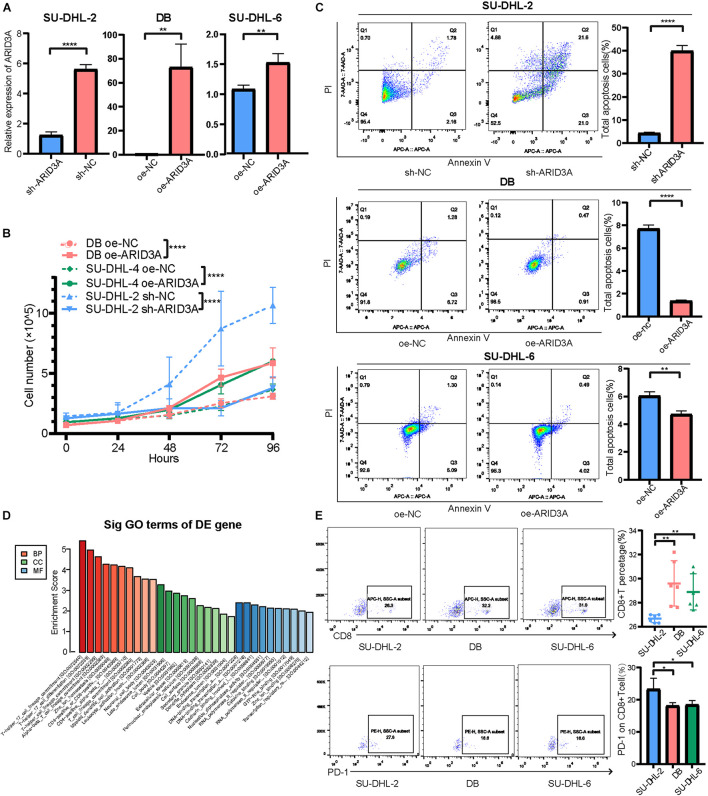
**(A)** Inhibition and overexpression of ARID3A in DLBCL cells was confirmed with RT-qPCR assays. **(B)** Cell proliferation ability of ARID3A knockdown SU-DHL-2 cells, ARID3A overexpressing DB and SU-DHL6 cells, and negative control cells was counted using trypan blue exclusion staining. **(C)** Cell apoptosis assays showing the effect of ARID3A knockdown on SU-DHL-2 cells and ARID3A overexpression on DB and SU-DHL-6 cell apoptosis. **(D)** Differentially intersected genes were subjected to GO term enrichment analyses. **(E)** CD8^+^ T cell percentage and the surface PD-1 expression was determined using flow cytometry analysis when co-cultured with DB and SU-DHL-2 cells and transfected cells. All data represented mean ± SD from three independent experiments. *P < 0.05, **P < 0.01; ****P < 0.0001.

Based on the above results, the high expression of miR-129-5p was considered a better prognostic factor for GCB subtype DLBCL compared to the ABC subtype. To further determine the specific mechanism, the ABC subtype SU-DHL-2 cells were transfected with miR-129-5p mimics and the GCB subtype DB and SU-DHL-6 cells with the miR-129-5p inhibitor. The results show that the proliferation ability of SU-DHL-2 cells was significantly stronger than that of DB and SU-DHL-6 cells. Meanwhile, overexpression of miR-129-5p impaired the proliferation ability of SU-DHL-2 cells. In contrast, inhibition of miR-129-5p promoted the proliferation of DB and SU-DHL-6 cells (Figure 3F). Cell apoptosis analysis shows that miR-129-5p overexpression in SU-DHL-2 cells resulted in a significantly increase in the number of apoptotic cells compared to control cells (8.29% vs. 5.38%, *P* = 0.0018), whereas knockdown of miR-129-5p in DB and SU-DHL-6 cells significantly decreased the percentage of apoptotic cells (7.14% vs. 3.03% and *P* = 0.0002, 7.89% vs. 3.22% and *P* < 0.0001, respectively, Figure 3G). We subsequently suppressed ARID3A expression levels in SU-DHL-2 cells and enhanced it in DB and SU-DHL-6 cells, which was verified by qRT-PCR (Figure 4A). The results show that the proliferation ability of SU-DHL-2 cells decreased and the number of apoptotic cells remarkably increased following ARID3A knockdown (4.34% vs. 39.97% and *P* < 0.0001). In addition, the overexpression of ARID3A in DB and SU-DHL-6 cells enhanced the proliferation ability and inhibited cell apoptosis (7.69% vs. 1.37% and *P* < 0.0001, 6.05% vs. 4.73% and *P* = 0.0036, respectively, Figures 4B,C). Together, these data suggest that downregulation of miR-129-5p and upregulation of ARID3A are responsible for the progression of ABC subtype DLBCL by promoting cell proliferation and inhibiting apoptosis.

### MiR-129-5p and ARID3A Affect the Interaction Between Diffuse Large B Cell Lymphoma Cells and CD8^+^ T Cells Through the PD-1/PD-L1 Immune Checkpoint

**FIGURE 5:**
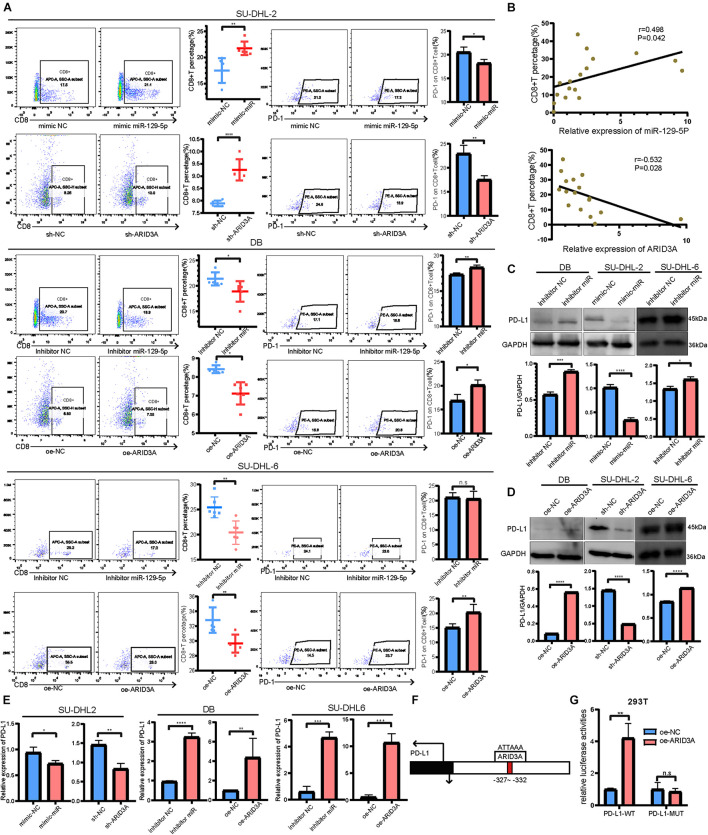
**(A)** CD8^+^ T cell percentage and the surface PD-1 expression were determined using flow cytometry analysis when co-cultured with SU-DHL-2, DB and SU-DHL-6 cells and transfected cells. Mean ± SD (*n* = 6). **(B)** Pearson’s correlation curve showing the positive relation between CD8^+^ T cells and miR-129-5p and the negative relation between CD8^+^ T cells and ARID3A in DLBCL peripheral blood. **(C,D)** Western blotting and densitometric analyses showing PD-L1 protein levels in transfected and negative control DB, SU-DHL-2 and SU-DHL-6 cells. **(E)** Expression of PD-L1 mRNA in transfected and negative control cells were determined using RT-qPCR. **(F)** Predicted ARID3A binding site in the PD-L1 promoter were acquired using JASPAR. **(G)** Dual-luciferase reporter gene assays were performed to show that ARID3A regulated PD-L1 as a transcription factor. Mean ± SD (*n* = 3). *P < 0.05; **P < 0.01; ***P < 0.001; ****P < 0.0001.

To further explore the downstream mechanisms of miR-129-5p and ARID3A, enrichment analysis of DIGs was conducted based on the Gene Ontology (GO) using DAVID (Huang da et al., 2009). As a result (Figure 4D), the top GO terms included T-helper 17 cell differentiation and CD4-positive or CD8-positive alpha-beta T cell lineage commitment, among others, which implies that these genes are closely related to the immune response. As reported, PD-L1 on tumor cells interferes with CD8^+^ T cell activity when communicating with PD-1 in the tumor microenvironment (Zheng et al., 2019). Therefore, we speculated that miR-129-5p and ARID3A could alter CD8^+^ T cells in DLBCL by changing the expression of PD-L1. To simulate the immune environment, DLBCL cells were co-cultured with immune cells. The results demonstrate that the proportion of CD8^+^ T cells co-cultured with SU-DHL-2 cells was significantly lower than that co-cultured with DB and SU-DHL-6 cells. And the expression of PD-1 on CD8^+^ T cells is higher than that co-cultured with GCB-subtype DLBCL cells. Thus, SU-DHL-2 cells have a stronger immune escape ability (Figure 4E). Moreover, knockdown of miR-129-5p or ectopic expression of ARID3A in DB and SU-DHL-6 cells reduced the percentage of CD8^+^ T cells and increased the PD-1 expression on the surface of CD8^+^ T cells, however, SU-DHL-6 failed to affect the PD-1 expression (Figure 5A). In contrast, the number of CD8^+^ T cells was significantly increased and PD-1 expression reduced after overexpression of miR-129-5p or knockdown of ARID3A in SU-DHL-2 cells. Furthermore, a significant association between CD8^+^ T cells and miR-129-5p or ARID3A expression was observed in patients with DLBCL (Figure 5B). A high level of miR-129-5p was associated with a higher number of peripheral blood CD8^+^ T cells (*P* = 0.042, *r* = 0.498). Conversely, low ARID3A expression was significantly correlated with a high CD8^+^ T cell ratio (*P* = 0.028, *r* = −0.532). Additionally, overexpression of miR-129-5p or silencing of ARID3A reduced the protein and mRNA levels of PD-L1 in SU-DHL-2 cells (Figures 5C–E). Inhibition of miR-129-5p or overexpression of ARID3A increased the protein and mRNA levels of PD-L1 in DB and SU-DHL-6. Next, we explored the regulatory mechanism of ARID3A on PD-L1 expression. ARID3A, a member of the ARID3 family, is a well-known transcription factor that can transcriptionally regulate gene expression by binding to the corresponding DNA sites (Tang et al., 2020). Therefore, we investigated whether ARID3A regulates PD-L1 expression at the transcriptional level. The ARID3A binding site of the PD-L1 promoter was obtained using the JASPAR tool (Figure 5F). Dual-luciferase reporter gene assay results show that the PD-L1 promoter transcription was increased after ARID3A overexpression, and this effect was significantly reversed by mutating the binding site (Figure 5G). In summary, it was validated that ARID3A, which is negatively regulated by miR-129-5p, can upregulate PD-L1 as a transcription factor.

### MiR-129-5p and ARID3A Targeted the Janus Kinase-Signal Transducer and Activator of Transcription Pathway

**FIGURE 6:**
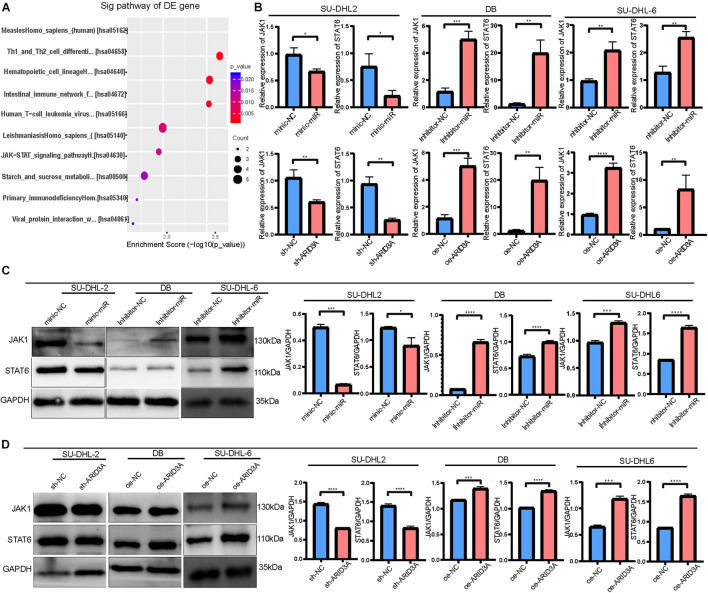
MiR-129-5p and ARID3A target the Janus kinase-signal transducer and activator of transcription (JAK-STAT) signaling pathway. **(A)** Differentially intersected genes were subjected to KEGG pathway analysis. **(B)** JAK1 and STAT6 mRNA levels in transfected DB, SU-DHL-2 and SU-DHL-6 cells were determined using RT-qPCR. **(C,D)** Western blotting and densitometric analyses showing JAK1 and STAT6 protein levels in transfected DLBCL cells. All data represented mean ± SD from three independent experiments. *P < 0.05, **P < 0.01; ***P < 0.001; ****P < 0.0001.

To further explore the differences between the ABC and GCB subtypes, DIGs were subjected to KEGG pathway analysis. Among them, the Janus kinase-signal transducer and activator of transcription (JAK-STAT) signaling pathway, associated with aggressive growth, invasion, and tumor-mediated immunosuppression, attracted our attention (Figure 6A). Therefore, JAK1 and STAT6 mRNA and protein levels were detected in DLBCL cells. As shown in Figure 6B, JAK1 and STAT6 mRNA levels in DB and SU-DHL-6 cells significantly augmented via inhibition of miR-129-5p and ARID3A overexpression. Meanwhile, Figures 6C,D demonstrate that miR-129-5p inhibitor and ARID3A overexpression significantly increased the protein expression of JAK1 and STAT6 in DB and SU-DHL-6 cells. In addition, both JAK1 and STAT6 were effectively reduced in miR-129-5p mimics or sh-ARID3A SU-DHL-2 cells compared with the negative control group. Our results indicate that miR-129-5p/ARID3A regulates DLBCL cell proliferation, apoptosis, and immune escape through the JAK-STAT signaling pathway.

### Relevance of Genes to Overall Survival

**FIGURE 7:**
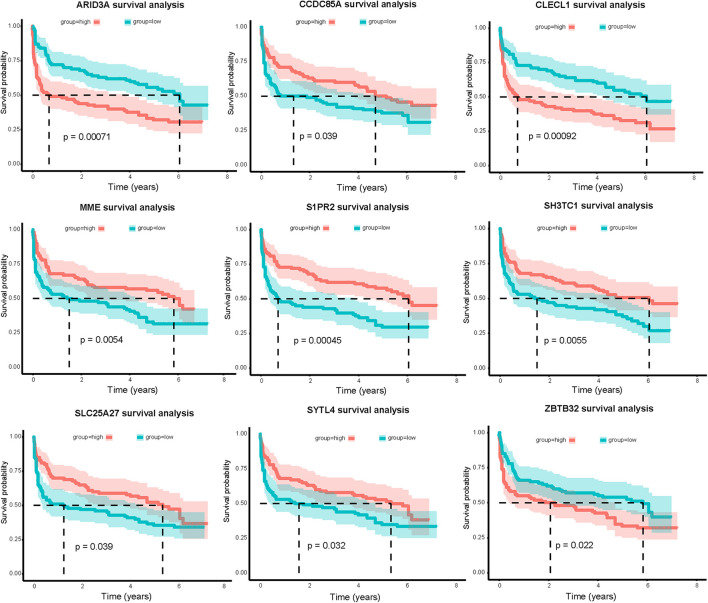
Nine mRNAs in the miRNA-mRNA regulatory networks are significantly related to overall survival of DLBCL patients in the GSE32918 database. Red and green lines indicate high-expression and low-expression groups, respectively.

Combining clinical information and gene expression information in GSE32918, 39 DEGs in miRNA-mRNA regulatory networks were evaluated using Kaplan-Meier survival analysis. As a result, the expression of nine mRNAs was significantly correlated with OS (*P* < 0.05, Figure 7). Surprisingly, we found a significant correlation between high ARID3A expression and poor prognosis in DLBCL patients. Therefore, we believe that ARID3A is an important prognostic and therapeutic target for DLBCL.

## Discussion

DLBCL is mainly divided into two COO subtypes, the GCB and ABC subtypes, with obvious differences in gene expression profiles and clinical outcomes. However, R-CHOP is the standard treatment for newly diagnosed DLBCL, regardless of the COO subtype. Although the combination of chemotherapy and rituximab result in a 5-year progression-free survival of 70–75% and OS of 75–80% (Cunningham et al., 2013), some patients, especially of the ABC subtype, still experience a high rate of relapse or refractoriness. Therefore, targeting strategies that are concentrated on different subtypes should be considered.

In recent years, miRNA expression profiles have been identified in a variety of cancers, including non-small-cell lung cancer (Niemira et al., 2019), breast cancer (Dastmalchi et al., 2020), and gliomas (Zeng et al., 2018), including DLBCL. However, the involvement of miRNAs in the pathogenesis and therapeutic response of different DLBCL subtypes needs to be further clarified. To identify the differential biomarkers of ABC and GCB subtypes, differentially expressed miRNAs and targeted genes between the two subtypes were identified based on one miRNA expression dataset and two gene expression profiles. Through bioinformatics analysis, a module with a strong correlation with a better prognosis of GCB subtypes was identified. By constructing a regulatory network between the MIGs and differential miRNAs, we identified three important miRNAs with the most targeted genes. Through the validation of DLBCL patient samples and cells, only miR-129-5p was significantly upregulated in the GCB subtype.

Studies have reported that miR-129-5p can inhibit the progression of rectal cancer (Wan et al., 2020), nasopharyngeal cancer (Yu et al., 2020), and liver cancer (Li Z. et al., 2019), but its role in DLBCL has not yet been reported. Our study found that miR-129-5p inhibited the proliferation and promoted apoptosis of DLBCL cells. This may be the reason why ABC-the subtype DLBCL with a low expression of miR-129-5p has a higher degree of malignancy. In addition, through bioinformatics analysis and *in vitro* experiments, miR-129-5p was found to negatively regulate ARID3A, which was confirmed by a luciferase reporter assay. As a transcription factor, ARID3A participates in many cellular processes and is reported to promote the progression of tumors such as ovarian cancer, colorectal cancer, and nasopharyngeal cancer (Dausinas et al., 2020; Tang et al., 2020). Our study demonstrated that ARID3A was highly expressed in ABC subtype DLBCL patients and cell lines, and the overexpression of ARID3A facilitated the proliferation of GCB DLBCL cells and inhibited cell apoptosis. The results of survival analysis based on the GEO dataset suggested that DLBCL with high ARID3A expression had a shorter survival time. Therefore, we believe that the high expression of ARID3A promotes the progression of DLBCL and leads to poor prognosis of ABC subtype DLBCL. Through KEGG pathway analysis of DEGs and *in vitro* experimental validation, we revealed that knocking down miR-129-5p and overexpression of ARID3A both resulted in increased expression of JAK1 and STAT6. An increasing body of evidence demonstrates that the JAK/STAT signaling pathway is an important target for tumor therapy because of its important role in aggressive growth, invasion, treatment resistance, and tumor-mediated immunosuppression (Ou et al., 2021). In addition, activation of the JAK/STAT signaling pathway is related to a worse prognosis in various cancers (Qureshy et al., 2020). Therefore, we suspected that tumor progression of the ABC subtype DLBCL is promoted through miR-129-5p/ARID3A-mediated activation of the JAK/STAT pathway.

GO function enrichment analysis indicates that genes were highly enriched in immune response-related signaling pathway. It has been reported that, in the tumor microenvironment, tumor cells can attenuate the activity of T cells and evade the immune response. Previous studies have pointed out that an increase in PD-L1 expression in tumor cells leads to a decrease in the proportion of CD8^+^ T cells that interact with it, thereby promoting tumor cell survival (Zheng et al., 2019). In the current study, miR-129-5p and ARID3A were found to alter the proportion of CD8^+^ T cells in the immune environment through PD-L1 activation, thereby promoting immune escape. We also show that ARID3A activated PD-L1 transcription by binding to its promoter. In this study, we first revealed that ARID3A, which is negatively regulated by miR-129-5p, activates PD-L1, leading to the immune escape of DLBCL, especially the ABC subtype.

However, there are some limitations in our study. First, the clinical sample size is too small, which may be one of the factors affecting our experimental data. Second, our experiments lack validation from animal experiments. Therefore, further studies are needed to elucidate the mechanisms of miR-129-5p and ARID3A.

## Conclusion

**FIGURE 8:**
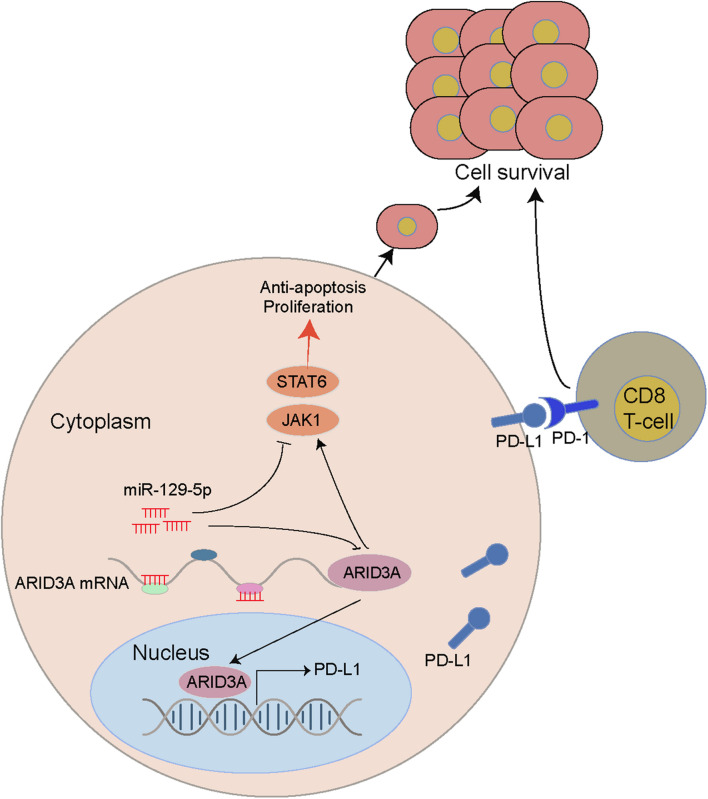
A miR-129-5P/ARID3A negative feedback loop modulates diffuse large B cell lymphoma progression and immune evasion through regulating the PD-1/PD-L1 checkpoint.

Through bioinformatics analysis and *in vitro* experiments, we revealed that miR-129-5p, a differentially expressed miRNA between the ABC and GCB subtypes of DLBCL, promoted apoptosis and inhibited proliferation and immune escape of DLBCL through targeted regulation of ARID3A. Specifically, we clarified that the miR-29-5P/ARID3A axis constitutes a negative feedback loop, which modulates PD-L1 expression in DLBCL and leads to changes in CD8^+^ T cell activity, ultimately promoting the immune escape of tumor cells (Figure 8).

## Data Availability Statement

The original contributions presented in the study are included in the article/Supplementary Material, further inquiries can be directed to the corresponding author/s.

## Ethics Statement

The study was approved by the Medical Ethics Committee of Fujian Medical University Union Hospital. The patients/participants provided their written informed consent to participate in this study.

## Author Contributions

WZ: conceptualization, methodology, investigation, original draft, review, and editing. GL: methodology, original draft, review, and editing. QL: original draft, review, and editing. MI: original draft, review, and editing. HF: review, editing, and supervision. JS: funding acquisition and supervision. All authors read and approved the final version of the manuscript.

## Conflict of Interest

The authors declare that the research was conducted in the absence of any commercial or financial relationships that could be construed as a potential conflict of interest.

## Publisher’s Note

All claims expressed in this article are solely those of the authors and do not necessarily represent those of their affiliated organizations, or those of the publisher, the editors and the reviewers. Any product that may be evaluated in this article, or claim that may be made by its manufacturer, is not guaranteed or endorsed by the publisher.

## Supplementary Material

Supplementary Figure 1 Gating strategy for the CD8^+^ T cell and PD-1-expressing CD8^+^ T cell. **(A)** Total lymphocytes were gated based on side-scatter and forward-scatter. **(B)** Doublets were eliminated from the analysis by FS-area (FS-A) and FS-height (FS-H). **(C)** Cells were further analyzed by expression of CD8. **(D)** Within CD8^+^ T cells and PD-1-expressing cells were identified.
