# Supplementary figures and images for: A miR-129-5P/ARID3A Negative Feedback Loop Modulates Diffuse Large B Cell Lymphoma Progression and Immune Evasion Through Regulating the PD-1/PD-L1 Checkpoint
Source: Front Cell Dev Biol. 2021 Oct 27;9:735855. doi: 10.3389/fcell.2021.735855 (PMC8579866; doi:10.3389/fcell.2021.735855)

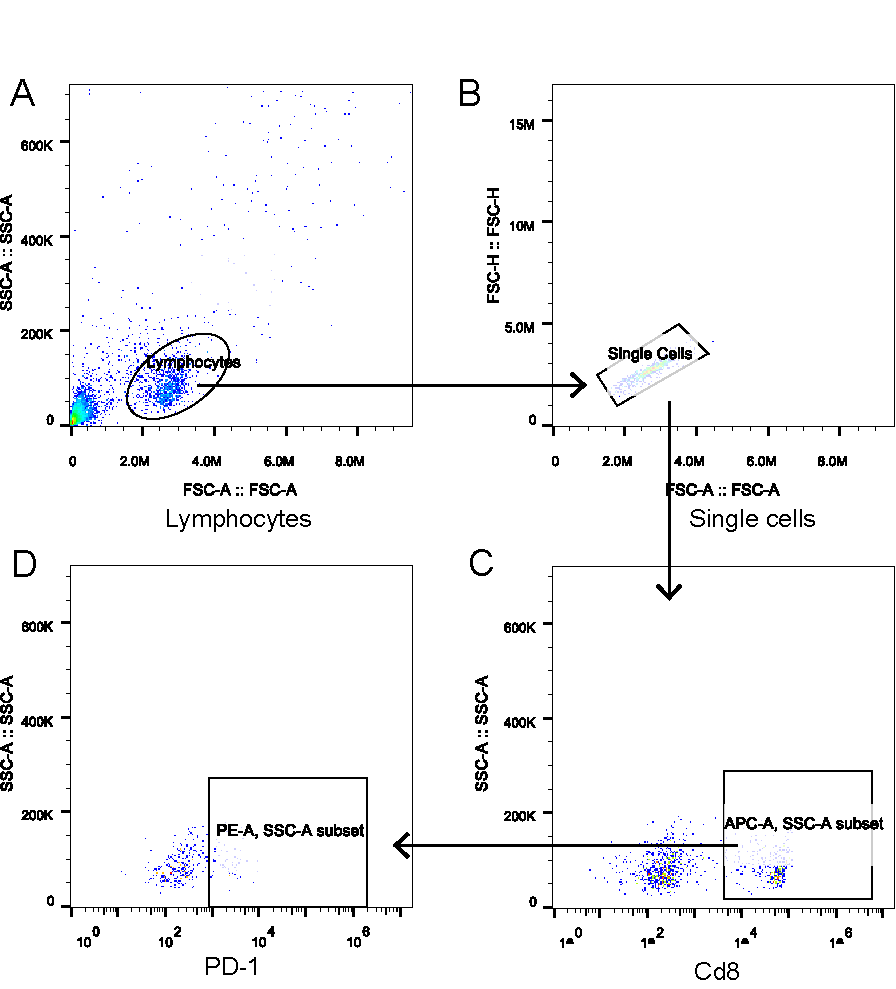

Supplement: Supplementary Figure 1 — Gating strategy for the CD8+ T cell and PD-1-expressing CD8+ T cell. (A) Total lymphocytes were gated based on side-scatter and forward-scatter. (B) Doublets were eliminated from the analysis by FS-area (FS-A) and FS-height (FS-H). (C) Cells were further analyzed by expression of CD8. (D) Within CD8+ T cells and PD-1-expressing cells were identified. [file Image_1.TIF]
