# Supplementary material for: A miR-129-5P/ARID3A Negative Feedback Loop Modulates Diffuse Large B Cell Lymphoma Progression and Immune Evasion Through Regulating the PD-1/PD-L1 Checkpoint
Source: Front Cell Dev Biol. 2021 Oct 27;9:735855. doi: 10.3389/fcell.2021.735855 (PMC8579866; doi:10.3389/fcell.2021.735855)
Supplement: Supplementary file 2 [file Table_1.docx]

supplemental Table S1

differentially intersected genes

| ACY3 |
| --- |
| ARL14 |
| ASB13 |
| ATP8A1 |
| BTNL9 |
| CCDC85A |
| CD1A |
| CILP |
| CPNE5 |
| CRHBP |
| CST1 |
| CUX2 |
| CYP39A1 |
| DEF8 |
| DNAJC5B |
| DNER |
| EEF1A2 |
| ENPP3 |
| FCER2 |
| FGD6 |
| GBA3 |
| GDPD5 |
| GNG8 |
| GPR82 |
| HIP1R |
| IQCD |
| KCNK12 |
| LRMP |
| LTBP1 |
| LY9 |
| MAML3 |
| MAP2 |
| MAPK10 |
| MARCKSL1 |
| MEF2C |
| MME |
| MMP7 |
| MYEOV |
| NLRP11 |
| NLRP4 |
| P2RY8 |
| PLAG1 |
| PLEKHF2 |
| PXDNL |
| S1PR2 |
| SERPINA9 |
| SH2B2 |
| SLAMF1 |
| SLC15A2 |
| SLC25A27 |
| SLC30A4 |
| SNX22 |
| SPINK2 |
| SSBP2 |
| STAG3 |
| STRBP |
| STXBP6 |
| SYTL4 |
| TEX9 |
| TIAM2 |
| TMEM119 |
| VPREB3 |
| WISP2 |
| ZBTB20 |
| ZNF318 |
| ZNF608 |
| AICDA |
| ARID3A |
| ARNT2 |
| BATF |
| BMF |
| C1orf186 |
| CCL22 |
| CCND2 |
| CCR10 |
| CHN2 |
| CLECL1 |
| COCH |
| CREB3L2 |
| CYB5R2 |
| EHHADH |
| ENTPD1 |
| FNBP1L |
| FUT8 |
| HLF |
| HPDL |
| IL12A |
| IL2RA |
| IRF4 |
| KCNA3 |
| KISS1R |
| KLHL21 |
| LILRA3 |
| MMP12 |
| MNDA |
| MT1H |
| MT2A |
| NETO2 |
| NLRP2 |
| PDLIM1 |
| PIM1 |
| PKN3 |
| PTGDR |
| PTPLA |
| RAVER2 |
| S100A8 |
| SFTPB |
| SH3TC1 |
| SLC38A5 |
| TNFRSF13B |
| TUBB6 |
| XK |
| ZBTB32 |
| ZNF385C |
| ZYG11A |
