# Supplementary material for: A miR-129-5P/ARID3A Negative Feedback Loop Modulates Diffuse Large B Cell Lymphoma Progression and Immune Evasion Through Regulating the PD-1/PD-L1 Checkpoint
Source: Front Cell Dev Biol. 2021 Oct 27;9:735855. doi: 10.3389/fcell.2021.735855 (PMC8579866; doi:10.3389/fcell.2021.735855)
Supplement: Supplementary file 3 [file Table_2.docx]

supplemental Table S2

modular intersection genes

| ARL14 |
| --- |
| ATP8A1 |
| CCDC85A |
| CRHBP |
| CYP39A1 |
| DNER |
| ENPP3 |
| IQCD |
| LTBP1 |
| MAML3 |
| MAP2 |
| MME |
| MYEOV |
| NLRP11 |
| NLRP4 |
| S1PR2 |
| SERPINA9 |
| SLC25A27 |
| SLC30A4 |
| SPINK2 |
| STAG3 |
| STXBP6 |
| SYTL4 |
| TEX9 |
| ARID3A |
| BATF |
| CCL22 |
| CCND2 |
| CLECL1 |
| COCH |
| CREB3L2 |
| ENTPD1 |
| HPDL |
| KCNA3 |
| KLHL21 |
| MMP12 |
| NETO2 |
| PTGDR |
| RAVER2 |
| SH3TC1 |
| TNFRSF13B |
| ZBTB32 |
