# Supplementary material for: A miR-129-5P/ARID3A Negative Feedback Loop Modulates Diffuse Large B Cell Lymphoma Progression and Immune Evasion Through Regulating the PD-1/PD-L1 Checkpoint
Source: Front Cell Dev Biol. 2021 Oct 27;9:735855. doi: 10.3389/fcell.2021.735855 (PMC8579866; doi:10.3389/fcell.2021.735855)
Supplement: Supplementary file 4 [file Table_3.docx]

supplemental Table S3

| miRNA | log2FC | P Value | Target genes |
| --- | --- | --- | --- |
| hsa-miR-107 | 1.72 | 0.0000 | ATP8A1/MYEOV/ENTPD1/ |
| hsa-miR-129-5p | 0.23 | 0.0780 | MME/ARID3A/MAP2/DNER/CYP39A1/LTBP1/COCH/CREB3L2/PTGDR |
| hsa-miR-140-3p | 0.17 | 0.2464 | PTGDR/DNER/ATP8A1/ENTPD1/MAP2/SYTL4/STXBP6/ |
| hsa-miR-140-5p | 1.32 | 0.0000 | ENTPD1/NLRP11/TEX9/SLC25A27/ENPP3//KCNA3/ |
| hsa-miR-142-3p | 0.26 | 0.5634 | ENTPD1/MAP2/NETO2/SYTL4/ENPP3/CREB3L2/ |
| hsa-miR-142-5p | 2.76 | 0.0000 | SLC30A4/MAP2/DNER/CYP39A1/LTBP1/NETO2/TEX9/CCDC85ASTXBP6 |
| hsa-miR-146b-3p | -0.70 | 0.0095 | ATP8A1/ARID3A/CCND2/ |
| hsa-miR-146b-5p | -0.70 | 0.0095 | SLC25A27/SYTL4/ARID3A/ |
| hsa-miR-193a-5p | -0.62 | 0.0047 | NETO2/CREB3L2/MAP2/DNER/ENPP3/ |
| hsa-miR-198 | -0.86 | 0.0023 | ZBTB32/RAVER2/CREB3L2/ENPP3/SLC30A4/CCND2/ |
| hsa-miR-199a-5p | 1.66 | 0.0003 | CREB3L2/MAP2/NETO2/CLECL1/TEX9/SYTL4/CCDC85A/SLC30A4/ |
| hsa-miR-199b-5p | 1.65 | 0.0084 | CREB3L2/MAP2/NETO2/CLECL1/TEX9/SYTL4/ENPP3/CCDC85A/SLC30A4 |
| hsa-miR-28-3p | 1.84 | 0.0000 | SYTL4/TEX9/ENPP3/ |
| hsa-miR-28-5p | -1.05 | 0.0000 | ATP8A1/SYTL4/TEX9/ |
| hsa-miR-296-3p | 2.12 | 0.0000 | NETO2/HPDL/ |
| hsa-miR-298 | -1.67 | 0.0000 | DNER/SYTL4/MME/NETO2/CCDC85A/ENPP3/SLC30A4/ |
| hsa-miR-299-5p | 0.18 | 0.3226 | MMP12/MAP2/SLC30A4/KCNA3/ |
| hsa-miR-300 | -1.27 | 0.0000 | CCND2/LTBP1/TEX9/CCDC85A/ |
| hsa-miR-331-3p | 0.05 | 0.5105 | CREB3L2/ |
| hsa-miR-338-3p | 0.13 | 0.1760 | MAP2/SYTL4/ |
| hsa-miR-342-3p | -0.24 | 0.3090 | DNER/NETO2/ |
| hsa-miR-361-3p | 0.79 | 0.0000 | KLHL21/ARID3A/S1PR2/SYTL4/ |
| hsa-miR-361-5p | 0.79 | 0.0000 | MAP2/COCH/CREB3L2/STXBP6/ENTPD1/ |
| hsa-miR-362-3p | 0.79 | 0.0019 | SYTL4/MAP2/NETO2/PTGDR/CCDC85A/ |
| hsa-miR-422a | -0.73 | 0.0477 | ATP8A1/TEX9/CCDC85A/SLC30A4/ |
| hsa-miR-485-3p | 0.47 | 0.0349 | CCDC85A/CRHBP/ENPP3/CREB3L2/MAML3/ |
| hsa-miR-490-3p | 0.00 | 0.5076 | CLECL1/SERPINA9/ENPP3/ |
| hsa-miR-491-3p | -1.50 | 0.0024 | CCDC85A/MME/CYP39A1/ |
| hsa-miR-509-5p | -0.96 | 0.0001 | ATP8A1/RAVER2/SLC25A27/SYTL4/SLC30A4/ENPP3/ |
| hsa-miR-515-5p | 0.73 | 0.0160 | LTBP1/ENTPD1/RAVER2/SYTL4/TEX9/PTGDR/ENPP3/ |
| hsa-miR-518a-5p | -0.73 | 0.0024 | ENTPD1/MAP2/RAVER2/SH3TC1/SLC30A4/ |
| hsa-miR-520d-5p | -0.95 | 0.0000 | ATP8A1/CRHBP/SLC30A4/LTBP1/TEX9/PTGDR/STXBP6/ |
| hsa-miR-525-5p | -0.68 | 0.0000 | MAP2/PTGDR/ENPP3/ |
| hsa-miR-574-5p | -0.34 | 0.0605 | MAP2/DNER/RAVER2/SYTL4/ARID3A/ |
| hsa-miR-575 | -0.61 | 0.0005 | MAP2/ |
| hsa-miR-576-3p | -1.04 | 0.0031 | MAML3/SYTL4/CREB3L2/CCDC85A/ |
| hsa-miR-583 | -1.38 | 0.0000 | SLC25A27/CREB3L2/ENTPD1/SERPINA9/ATP8A1/SYTL4/ENPP3/ |
| hsa-miR-590-5p | 0.08 | 0.1705 | ENTPD1/LTBP1/NETO2/CCL22/CCDC85A/ |
| hsa-miR-617 | -0.74 | 0.0001 | KLHL21/DNER/ |
| hsa-miR-620 | -1.10 | 0.0007 | CCL22/ENTPD1/DNER/ |
| hsa-miR-623 | -0.69 | 0.0253 | CCND2/CRHBP/SLC25A27/ENPP3/SH3TC1/CCDC85A/KCNA3/ |
| hsa-miR-628-3p | -0.98 | 0.0000 | SYTL4/ARL14/ |
| hsa-miR-630 | -0.66 | 0.0002 | MME/ |
| hsa-miR-634 | -0.66 | 0.0008 | CCL22/KLHL21/SYTL4/CCND2/ |
| hsa-miR-635 | -0.92 | 0.0010 | KCNA3/SLC25A27/SYTL4/ |
| hsa-miR-636 | 0.64 | 0.0084 | CRHBP/ |
| hsa-miR-637 | -1.57 | 0.0012 | S1PR2/CREB3L2/ZBTB32/STAG3/PTGDR/CCDC85A/ |
| hsa-miR-649 | 1.34 | 0.0000 | MAP2/ATP8A1/CCL22/ |
| hsa-miR-658 | -1.04 | 0.0000 | S1PR2/STXBP6/ |
| hsa-miR-665 | -1.25 | 0.0069 | ATP8A1/SPINK2/ZBTB32/STAG3/SH3TC1/TNFRSF13B/CCL22/CCND2 |
| hsa-miR-765 | -1.04 | 0.0000 | MYEOV/CREB3L2/STXBP6/ |
| hsa-miR-920 | -0.91 | 0.0020 | ATP8A1/NETO2/CCL22/CCDC85A/ENTPD1/SLC30A4/ |
| hsa-miR-921 | -0.88 | 0.0000 | COCH/ |
| hsa-miR-933 | -0.83 | 0.0056 | CRHBP/ |
| hsa-miR-934 | -0.68 | 0.0034 | SLC25A27/DNER/ATP8A1/CYP39A1/ |
| hsa-miR-943 | -0.90 | 0.0244 | CCDC85A/ATP8A1/ |
